# Sequential Folding using Light-activated Polystyrene Sheet

**DOI:** 10.1038/srep16544

**Published:** 2015-11-12

**Authors:** Yonghee Lee, Hyeok Lee, Taesoon Hwang, Jong-Gu Lee, Maenghyo Cho

**Affiliations:** 1 WCU Multiscale Mechanical Design Division, School of Mechanical and Aerospace Engineering, Seoul National University, Seoul, 151-144, Korea

## Abstract

A pre-strained polystyrene (PS) polymer sheet is deformed when it approaches the glass transition state as a result of light absorption. By controlling the light absorption of the polymer sheet, non-contact sequential folding can be accomplished. Line patterns of different transparencies and shapes are used to control the light absorption. The line pattern shape is closely related to the folding angle and folding start time. The relation between the line pattern design and folding performance was evaluated experimentally to develop a technique for folding PS sheets. The results show that sequential folding of PS sheets can be accomplished by changing the degree of transparency of the line pattern. Using the technique developed in this study, self-folding origami structures with complicated shapes can be designed and manufactured.

Light-activated self-folding has recently been investigated to assess its usefulness in a variety of applications, such as robotic systems^1^,^2^,^3^,^4^,^5^. The self-folding technique is considered to be an effective method of influencing the behaviour of complex shapes on the mesoscale. This technique can be applied to origami systems for three-dimensional deformed shapes, such as activation devices for robotic systems^6^,^7^,^8^, MEMS^9^,^10^, biomedical devices^11^, solar power cells^12^, sensors^13^, and drug delivery systems^14^. Self-folding can be accomplished in several ways. One of the most commonly used self-folding techniques involves the use of shape-memory material^15^,^16^,^17^,^18^,^19^,^20^,^21^,^22^. Of course, folding of patterned composites using stiffness differences^23^ or electrical devices with metalized polyester film (MPF)^24^ is a well-known self-folding technique. Polystyrene (PS) polymer sheets have recently been identified as materials that are well suited for light-activated self-folding. PS polymer is a frequently utilized material that is readily available, has adequate stiffness, is low in cost, is environmentally friendly, and has good transparency^25^,^26^,^27^,^28^,^29^. In the case of PS sheets manufactured by applying pressure and heat, such as Shrinky Dinks product, thermal contraction of a PS sheet occurs when the sheet is heated above a specific temperature (the glass transition temperature, which is approximately 102 °C) and it approaches the glass transition state. While the thermal contraction of PS polymer at the glass transition state makes it useful in other research fields, such as manufacture of microstructure^30^, its usefulness in light-activated folding is attributable to its transparency. If thermal energy is delivered by infrared rays, most of them will pass through a transparent PS sheet. However, if black-coloured lines are printed on a PS sheet, selective light absorption by the line pattern is possible^1^,^2^,^3^. The upper surfaces of the black-coloured lines approach the glass transition state more rapidly than the bottom surfaces, so localized contraction occurs. This contraction gap in a cross section of a PS sheet causes an extreme deformation manifested as folding behaviour. This folding behaviour can be induced easily by printing a black line pattern on a PS sheet and applying light. Unlike other self-folding techniques, this technique for inducing folding behaviour in a PS sheet has the distinctive characteristic that it is activated by light. This technique does not require a direct connection to a power source because the folding behaviour of the PS sheet is based on the application of light, including infrared rays. Other advantages of this technique are that light is an eco-friendly energy source that is not harmful to humans and that the low production cost of PS sheets is conducive to widespread use of the light-activated self-folding technique.

The use of folding to control pre-straining of polymer sheets has been examined in previous research^4^. Ryu *et al.* manufactured a locally pre-strained polymer sheet and illuminated the polymer sheet to make it deform to a specific folded shape. While direct design of polymer sheets has various advantages, such as control of the material characteristics and geometric shape, the manufacture of locally pre-strained polymer sheets requires considerable skill and dexterity. In contrast, PS sheets with line patterns can be manufactured easily by anyone who can use a desktop printer, so PS sheets with line patterns can be produced more readily than locally pre-strained sheets. Accordingly, PS sheets with line patterns were employed in this study to examine the light-activated self-folding technique. Furthermore, using combinations of folds, self-folding origami structures can be developed. Self-folding origami structures such as octahedrons and icosahedrons can be designed by varying the line patterns printed on planar PS sheets. However, a PS sheet might not be deformed to the intended shape, because its folding behaviour is based on the thermal contraction of the polymer material, and the instability of this deformation behaviour makes it difficult to produce the intended shapes precisely. As an alternative, collar faces are considered. A collar face is an additional face attached at the edge line of the PS sheet. When a PS sheet with many line patterns is folded, the collar face is in contact with the other faces. Through this process, the collar face prevents excessive folding deformation, which makes the PS sheet maintain the desired folding angle. In addition, to prevent collisions between collar faces, the sheets are sequentially folded. The light absorption of a PS sheet is controlled by changing the degree of transparency of the line pattern, so sequential folding can be achieved through control of the degree of transparency of the line pattern.

Because the self-folding behaviour of PS sheets is a recently discovered phenomenon, only a few experiments on the folding properties of these sheets have been performed^1^,^2^,^3^, and sequential folding of PS sheets has not been studied yet. Therefore, this study was conducted to examine the properties of PS sheets that influence sequential folding along line patterns experimentally. The experiment was focused specifically on examining the relationship between the line pattern and the folding behaviour of a PS sheet, because the critical factor in the folding behaviour of a PS sheet is the black line pattern printed on the sheet. Hexahedral, octahedral, and icosahedral structures were selected as examples of self-folding structures. A simple shape such as a hexahedron can easily be achieved with simple line pattern designs. In contrast, it is almost impossible to form more complex shapes such as icosahedrons by simultaneous folding. Accordingly, a sequential folding technique that takes advantage of differences in the degrees of transparency of the lines is employed, and a collar face is applied to the edge line of the PS sheet to achieve stable folding deformation.

The influence of heat should be considered in a plastic model of a PS sheet because the folding behaviour of the PS sheet is based on contraction in response to thermal absorption. However, the deformation of a PS sheet starts at the glass transition state under stress-free conditions, so the model must represent the strain present at a specific temperature without any external force being applied. Accordingly, the plastic curve of the PS sheet is changed by the absorption of heat. When the sheet passes the glass transition temperature as a result of heat absorption, the *x*-intercept of the plastic curve occurs. Thus, strain can be generated at the glass transition state without the application of external force.

In this paper, the sequential folding behaviour of PS sheets using the degree of transparency of line pattern was studied for use in the design and manufacture of self-folding origami structures. Sequential folding is a novel part of the work, by which we obtain complex self-folding structure of polystyrene polymer. Experiments in light-activated folding deformation of PS sheets were conducted to assess their folding properties. Based on the results of the experiments, we developed a process for the fabrication of three-dimensional complex folding structures from flat PS sheets.

## Results

### Folding behaviour of a PS sheet activated by light

Thermal contraction strain in a PS sheet is generated at the glass transition state without the application of any external force, but this phenomenon cannot be expressed by the usual plastic constitutive model. To describe the folding behaviour of a PS sheet at the glass transition state, the plastic model is modified to reflect deformation by heat. If the model is assumed as the linear elasto-plastic state, the yield function of the rotation angle with respect to the bending moment is expressed as follows:


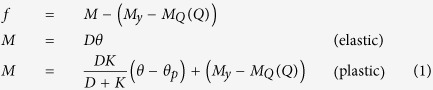


**Figure 1:**
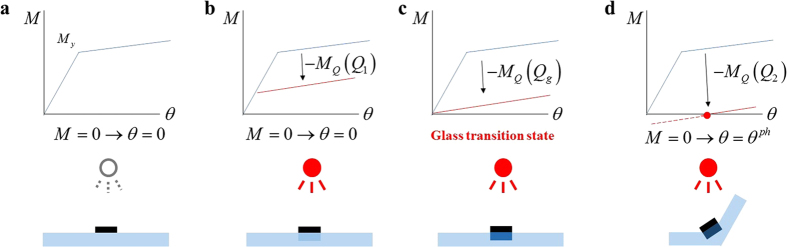
Rotation angle-bending moment curve changed by absorbed heat. (**a**) No-light state (no absorbed heat). (**b**) Initial lighting state (no folding behaviour due to non-glass transition state). (**c**) Glass transition state. (**d**) Folding occurrence due to absorbed heat without external force.

**Figure 2:**
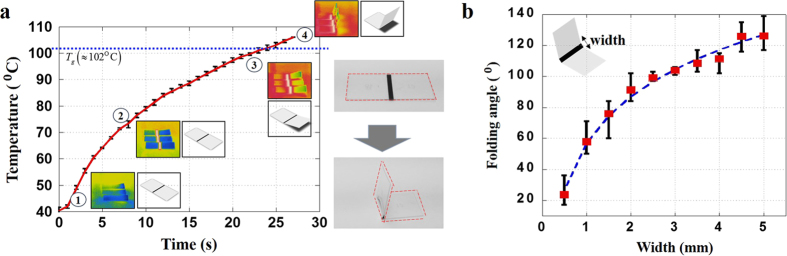
Folding deformation of the PS sheet when is exposed to light. (**a**) Temperature variation of the PS sheet. (**b**) Folding angle versus width of black line pattern of PS sheet.

In Eq. (1), the yield function *f* is expressed using the bending moment *M*, yield moment 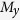, and yield moment variation term 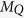, which is influenced by the heat *Q*. The bending moment *M* is defined differently in the elastic and the plastic state. In the elastic state, the bending moment is obtained from the bending modulus *D* and the rotation angle 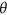. In the plastic state, the bending moment is expressed by adding the hardening modulus *K*, the plastic rotation hinge angle 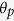, and the yield moment variation term 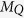. In the no-light irradiation state, the contraction of the PS sheet is not generated without force, as shown in Fig. 1a. When the PS sheet absorbs light, the temperature of the line pattern increases because of the heat produced by the light. However, folding does not occur below the glass transition temperature because not enough heat is absorbed by the PS sheet (Fig. 1b). At the glass transition state, the plastic curve approaches the origin point in Fig. 1c, and folding occurs when the temperature exceeds the glass transition state (Fig. 1d). As the employed constitutive model is influenced by the heat, the folding deformation at the glass transition state can be expressed in the analysis of the PS sheet. The PS sheet specimens used in this study were manufactured in sizes of 30×15 mm, with a pattern of lines spaced 1 mm apart and the transparency of 0% (black). In the thermographic images ①–④ in Fig. 2a, the black line pattern corresponds to the highest temperature because the black line pattern absorbs almost all of the light. Over time, the temperature of the line pattern increases, and folding occurs when the temperature of the line pattern approaches 102 °C, which is the glass transition temperature. However, the temperature of the unprinted area of the PS sheet increases slightly during the lighting process because light cannot penetrate the transparent parts of the PS sheet completely. In addition, the line pattern of the folded PS sheet produces contraction parallel to the line direction, and the conduction of heat from the line pattern to the transparent parts of the PS sheet is another factor that interferes with the folding behaviour. Accordingly, long-duration light exposure can hinder the folding behaviour of the PS sheet because of the contraction of the sheet as a whole. To assess the folding behaviour of the PS sheet that is associated with the line pattern, the duration of the exposure to light should therefore be limited. Accordingly, the maximum lighting time was set to 1 minute in the experiments described below.

The folding behaviour of PS sheets is induced by lighting, and the pattern of printed lines is directly related to how the PS sheet folds. The folding angle is the most important variable in the design of the folding behaviour. The folding angle is determined by the localized contracted area, as shown in Fig. 2b. The curve describing the folding angle as a function of line pattern width shows a more sharp increase than that of the model proposed by Liu *et al.*^3^. This is because of the thickness difference of the PS sheet. The PS sheet used in this study had a thickness 0.24 mm, thinner than that used in Liu’s model (0.3 mm). This thickness difference of PS sheet corresponds to a folding angle difference at each line pattern width. In this study, the line pattern width was varied from 0.5 mm to 5 mm in increments of 0.5 mm, and the degree of transparency was fixed at 0% (black). A bilinear curve was employed as the fitting curve because the folding angle increases rapidly for a specific line width. Using the least squares regression method, the following best-fit curves were obtained:


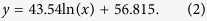


The folding behaviour of the PS sheet may not coincide entirely with the planned shape because of the thermal contraction parallel to the direction of the line pattern. In the narrow width range (0.5 mm–2 mm), the influence of the thermal contraction parallel to the line direction increases, and the Poisson effect at the line pattern causes the scattering of the observed folding angles. Additionally, when the line pattern is printed with a larger width (3.5 mm–5 mm), it may exhibit the non-uniform printing carbon density owing to the smooth surface of the PS sheet. This results in the scattering of the observed folding angles. Because of these reasons, the most stable folding behaviour is observed at a line width of 2.5–3 mm. While the folding angle is proportional to the line width, a thicker line pattern does not unconditionally guarantee a greater folding angle. The proper width range for successful folding of a PS sheet is influenced by the PS polymer material characteristics and the thickness of the sheet. A sheet of Shrinky Dinks was employed in this study, and an appropriate width range for the folding behaviour of this PS sheet was presumed to be 0–5 mm.

**Figure 3:**
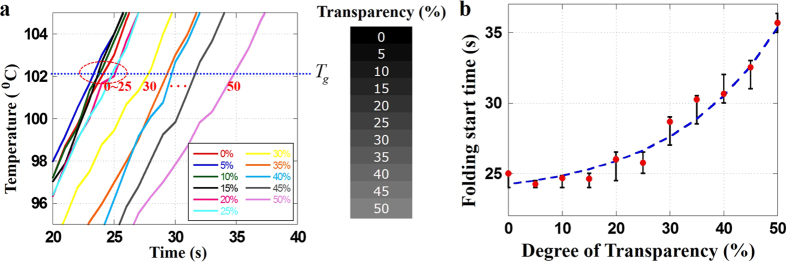
PS sheet folding behaviour versus line pattern transparency of PS sheet. (**a**) Temperature variation over time for various line pattern transparencies. (**b**) Folding start time versus line pattern transparency.

While control of the folding angle is critical to achieving the desired folding behaviour, sequential folding is another key point. Two methods can be used to accomplish sequential folding of a PS sheet. The first method is light control. As the light is absorbed to different degrees by the line pattern, the folding start times differ depending on the rate of temperature increase. Sequential folding by regulation of light exposure does not require modification of the line pattern, and the light can be controlled in the process of folding. Light control thus enables more precise control of the folding behaviour of the PS sheet. However, light control is not an easy technique, and it demands substantial equipment resources. The second method, which uses the transparency of the line pattern, can more easily achieve sequential folding. More varied and low-cost light absorption control is possible because the line pattern is printed using a desktop printer. Thus, the method that uses transparency variation of the line pattern was employed for sequential folding in this study. To assess the influence of line pattern transparency on the sequential folding design, the temperature variation around the line patterns over time was measured. The results are illustrated in Fig. 3.

The degree of transparency of the line pattern was varied from 0% to 50%, where 0% transparency corresponds to “black” and 100% transparency corresponds to “clear.” The line width of the line pattern was fixed at 2 mm. When light passes through a PS sheet with a grey-coloured line pattern, folding deformation occurs later than when light passes through a sheet with a black-coloured line pattern, because of the increase in light penetration. As the line pattern becomes more transparent, the nonuniform lack of clarity of the material interferes with the penetration of the light. Thus, the variation in the folding start time tends to increase as the degree of transparency of the line pattern increases. In addition, the relationship between the folding time and the degree of transparency exhibits a sharp increase at a specific point. The following bilinear curve was fitted to the data describing this phenomenon:


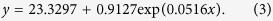


The dividing point was selected as the point with the smallest standard deviation. In addition, the time gap in seconds between folding lines is required for the sequential folding process, so the line pattern cannot have numerous divided sections. It is proposed that 4–5 sections be used.

### Self-folding origami structure design

**Figure 4:**
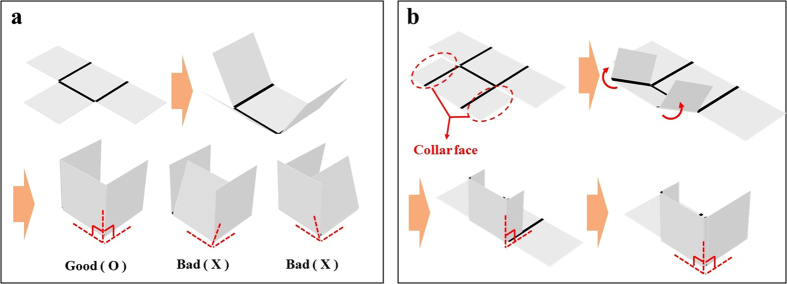
Stability of the collar face and sequential folding combination. (**a**) Unstable folding behaviour of the PS sheet without collar faces. (**b**) Stable folding behaviour of the PS sheet using collar faces.

Based on the folding properties of a PS sheet, the design of a self-folding origami structure is possible. The self-folding behaviour of a PS sheet with a few line patterns can be predicted easily, so the final design shape can be achieved without difficulty. However, a more complex self-folding origami structure, such as an icosahedral shape, requires careful design because of the nonuniform folding behaviour that must be induced by thermal conduction and contraction of parallel line patterns. If the faces of the PS sheet fold unexpectedly, it is difficult to obtain the planned shape as the final result. That is, slight deviations can prevent the achievement of the desired shape, as shown in Fig. 4a.

**Figure 5:**
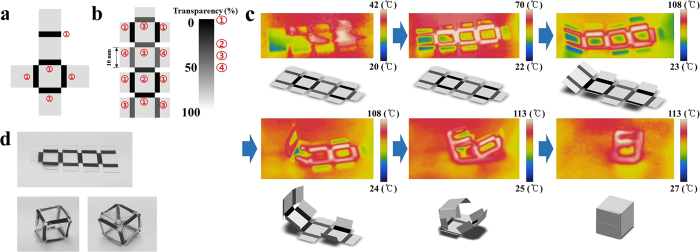
Hexahedral shape deformation of PS sheet. (**a**) Basic design of the hexahedral shape of the PS sheet. (**b**) Design using sequential folding of the PS sheet (**c**) Thermographic image of hexahedral shape deformation process. (**d**) Undeformed and deformed PS sheet for hexahedral shape.

To achieve more stable folding behaviour, a collar face at the edge of the PS sheet can be considered. The folding angle can thereby be maintained as planned because the collar face prevents excessive folding of the side faces. Therefore, a collar face on a PS sheet can ensure the stability of self-folding, as shown in Fig. 4b. In addition, sequential folding based on transparency can be employed for the folding sequence of line patterns. In this study, some models were manufactured as examples of self-folding origami structure designs. First, a hexahedral shape was designed using a PS sheet. If six faces are used, such a shape can be achieved easily with a uniform line pattern width and degree of transparency, as shown in Fig. 5a. However, folding using just six faces may produce an imperfect shape. Thus, collar faces were added to the edge line of the PS sheet, and different degrees of transparency were used, as shown in Fig. 5b. The folding angle of hexahedral shape is 90°, and the line pattern width is calculated to be 2.14 mm from Eq. (2). However, for the more stable folding behaviour, the line width is designed as a little thicker line width 2.3 mm, because a collar faces attached in PS sheet prevents excessive folding deformation.

In the folding process for the model shown in Fig. 5b, the top face of the final shape and the four collar faces were folded first, and the other faces were then folded in order. The line width was set to be sufficient to produce 90° folds, and slight overfolding was considered permissible because of the collar faces. The self-folding process for the PS sheet was observed using a thermographic camera. Thermal images of the folding process are shown in Fig. 5c. The folding deformations of the line patterns are not generated at the same time because the degrees of transparency of the line patterns are different for sequential folding, as shown in Fig. 5c. In the no-light state, the temperature is uniformly distributed. As light is applied to the PS sheet, the black-coloured line patterns start to show the highest temperatures. However, folding deformation does not occur when the highest temperature is lower than the glass transition temperature. Folding occurs along the 0% transparency line at the glass transition temperature, and folding occurs along other lines as they approach the glass transition state. The collar faces prevent excessive folding and disturbance by other faces so that stable folding behaviour into a hexahedral shape occurs. The final hexahedral shape is achieved by stable deformation of the PS sheet, as Fig. 5d shows.

**Figure 6:**
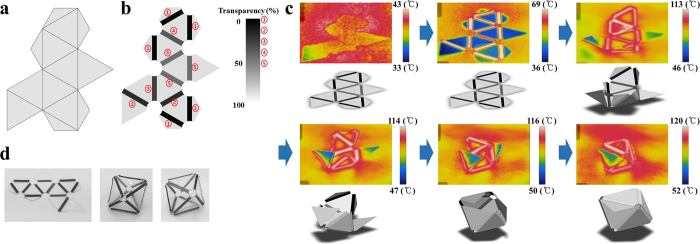
Octahedral shape deformation of PS sheet. (**a**) Planar figure for octahedral shape. (**b**) PS sheet design using sequential folding. (**c**) Thermographic image of octahedral shape deformation process. (**d**) Undeformed and deformed PS sheet for octahedral shape.

As a second model for self-folding origami structure design, a PS sheet for an octahedral shape was manufactured. Collar faces were added to the octahedral faces, as shown in Fig. 6a, and a pattern of lines of various degrees of transparency were printed along the fold line, as shown in Fig. 6b.

The degrees of transparency of the lines were set to control the folding sequence, and the intersection points of the lines were cut out to prevent excessive light absorption in densely coloured areas. While the folding angle (180° minus the dihedral angle) of a hexahedral shape is 90°, the folding angle of an octahedral shape is 71.53° (dihedral angle =109.47°). By decreasing the line pattern width to 1.5 mm, the folding angle of the octahedral shape was controlled. The PS sheet for the target (octahedral) shape is shown in Fig. 6d. As a result of light absorption, the undeformed PS sheet shown in Fig. 6d was deformed to the octahedral shape. Because the collar faces prevented excessive folding deformation, the PS sheet could be deformed to the intended shape. In addition, the folding deformation of the PS sheet into the octahedral shape could be examined using thermal images of the PS sheet folding process, as shown in Fig. 6c. In the thermal images of the octahedral shape deformation, folding begins along the black-coloured lines of the PS sheet at the glass transition state. Other line patterns are sequentially folded in accordance with the degrees of transparency. An octahedral shape is ultimately obtained by self-folding of the PS sheet.

**Figure 7:**
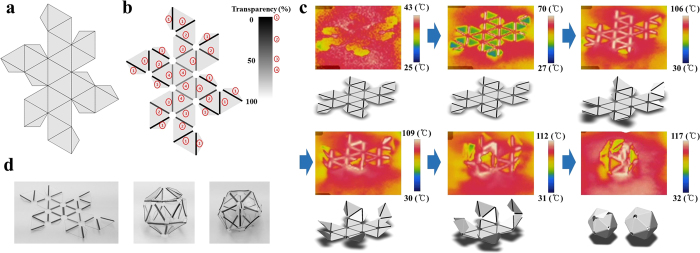
Icosahedral shape deformation of PS sheet. (**a**) Planar figure for icosahedral shape. (**b**) PS sheet design using sequential folding. (**c**) Thermographic image of icosahedral shape deformation process. (**d**) Undeformed and deformed PS sheet for icosahedral shape.

In the case of a hexahedral or octahedral shape, the folding behaviour needs to be precisely designed because it occurs simultaneously in several areas, despite sequential folding design through transparency control. Furthermore, precise control of the folding behaviour is required to produce an icosahedral shape by deformation of a PS sheet. An icosahedron, which consists of 20 faces, requires 19 line patterns without collar faces, and a sheet with 10 collar faces must have 10 additional line patterns. Therefore, careful design and manufacturing are required to fold a PS sheet into an icosahedron. The folding angle of an icosahedron is 42.81° (dihedral angle =137.19°), and the line pattern width is designed to 0.8 mm for the folding angle. In addition, the positioning of each of the 20 faces is particularly important in the design of a self-folding origami structure. Thus, in this study, for the icosahedron-shaped model of a self-folded PS sheet, the model shown in Fig. 7b was considered.

If the faces of an icosahedron are linearly connected, unlike those shown in Fig. 7a, the faces folded first may be deformed by excessive light absorption. Thus, the faces should be located in radial form with point symmetry. In addition, while a hexahedral or octahedral shape produced by deformation of a PS sheet can be achieved with comparative ease, formation of an icosahedral shape requires a more delicate line pattern design. This results in greater transparency variation and may result in a line pattern with high transparency that is folded under non-horizontal conditions because of the other folding line patterns present. Therefore, to avoid interaction between the line patterns, a black-coloured line pattern was located in the external areas of the sheet, and a grey-coloured line pattern was located within the interior of the PS sheet. The degrees of transparency range was expanded to 0–60%, as shown in Fig. 7b. In addition, the intersections between line patterns were cut out to prevent excessive light absorption, as in the case of formation of the octahedral shape, as described previously. The PS sheet used to produce the icosahedral shape and the deformed shape obtained are shown in Fig. 7d.

Like the hexahedral and octahedral shapes, deformation of a PS sheet into an icosahedral shape was observed using thermal images, as shown in Fig. 7c. The collar faces ensured that the PS sheet was folded into faces at the desired angle, and the desired icosahedral shape was obtained.

## Discussion and limitation

The self-folding structure using PS sheet is designed by printing black line patterns to PS sheet. While this technique is easily utilized, some limitations must be considered due to its characteristics that comes from light-absorption.

1. The light-absorption rate in the line pattern is very high, and the region of transparent sheet near line pattern absorbs a little light. In the course of time, the transparent area is contracted by light-absorption, and this contraction can disturb the folding behaviour of PS sheet. Accordingly, the time limit for the definite folding behaviour of PS sheet should be considered in the self-folding structure design, and the time limit is determined by the light intensity and room temperature.
2. The folding deformation of PS sheet is caused by the depth-dependent light absorption, and this is achieved by the black-coloured line patterns. The folding angle is controlled by line pattern width. However, the excessively wide line pattern disturbs depth-dependent light absorption, then overheated PS sheet is deformed to unexpected shape. Line pattern printed in the PS sheet should be made in a limited width.
3. The sequential folding of PS sheet is controlled by the degree of transparency of line pattern. Through light-absorption control using the degree of transparency, the each line pattern can be sequentially folded. However, it takes some time to complete the folding deformation of PS sheet, and the time from the start to complete the folding is important for the sequential folding. If the second line pattern starts to be folded after the completion of the first folding deformation, PS sheet is deformed to the planned shape. However, if the second line pattern is simultaneously folded while the first line pattern is being folded, their folding deformations can interrupt one another. Accordingly, the time gap in folding lines should be considered for stable sequential folding.

## Conclusion

We have demonstrated the light-activated sequential folding behaviour of PS sheets. As light absorption is concentrated in specific areas by black-coloured line patterns printed on PS sheets, localized contraction can be employed to induce folding behaviour. The self-folding deformation of a PS sheet can be activated by non-contact control through light absorption. The folding behaviour of a PS sheet can be controlled by varying the properties of the line pattern printed on the sheet, which can be done using a typical desktop printer. The folding behaviour is closely related to the line pattern printed on the PS sheet, as confirmed by the results of the experiments on the folding behaviour of PS sheets conducted in this study. Increasing the width and transparency of the line pattern increases the folding angle and folding time, respectively, and this relation can be utilized in self-folding origami structure design. However, although the folding behaviour of a PS sheet can be easily controlled, the design of PS sheets requires precise design and manufacturing techniques.

To achieve stable folding deformation of a PS sheet, the collar face at the edge line of the PS sheet needs to be considered. In addition, sequential folding using variable line transparency can be accomplished to prevent contact between collar faces and allow the PS sheet to be stably deformed. In this study, we measured the deformation properties of PS sheets, and we manufactured PS sheets for use in self-folding origami structure formation, such as hexahedral, octahedral, and icosahedral shapes. As mentioned earlier, because three-dimensional shape fabrication using PS sheets can be achieved easily, we consider the technique to be suitable for use in the fabrication of various three-dimensional structures based on the folding deformation mechanism.

## Methods

### Experimental Conditions

**Figure 8:**
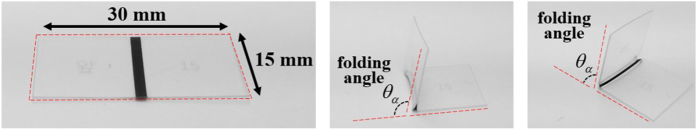
Polystyrene sheet for self-folding deformation experiment. (**a**) Undeformed specimen configuration. (**b**) Deformed specimen. (**c**) Folding angle of deformed PS sheet by light.

Precise folding deformation requires a well-manufactured PS sheet. Shrinky Dinks PS sheets were employed in this study because they exhibit good folding behaviour when exposed to light. The specimens had dimensions of 30 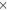 15 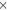 0.24 mm, and a pattern of black lines with widths of 0.5 to 5 mm were printed up to the mid-line of each specimen, as shown in Fig. 8.

The folding deformation time was measured for the degrees of transparency of 0% to 50%. Black lines were printed on PS sheets using a desktop laser printer (HP-CP 4050). During the folding deformation process, the temperature distribution of each PS sheet was observed using a thermographic camera (FLIR-E30). The real-time temperature variation of the PS sheets was observed, and the folding start time was measured. When each PS sheet was folded, the light intensity of the bulb and the distance between the specimen and the light bulb were controlled. For uniform the light intensity distribution, the lamp was located at a reasonable distance (17 cm). Six linear halogen lamps (OSRAM J118-OS, 300W), arranged in parallel, served as the light source. If the target structure was intended to be a simple shape, the light intensity did not need to be powerful because there are few fold lines in a simple shape. However, in the case of complex geometries such as icosahedral shapes, excessive heat can be concentrated in the centre area, so the lighting time must have a definite limit. The light intensity should therefore be maintained to achieve sufficient folding along the line patterns in a sufficiently short time. In this study, the light intensity was fixed as 400 mW/cm^2^.

The folding behaviour of a PS sheet depends on its thermal deformation and therefore on the surrounding temperature. The importance of surrounding temperature in PS sheet folding deformation is researched in previous studies^1~3^. If the PS sheet is located in an environment with a temperature of 90 °C, a temperature increase of just 12 °C is required for light-activated deformation. A PS sheet in an environment of 60 °C area requires much more thermal energy than one in an environment of 90 °C. Accordingly, the surrounding temperature can be a critical factor in the self-folding design behaviour of a PS sheet. Sequential folding takes advantage of light absorption differences arising from differences in the transparency of the black lines in the printed pattern used, so a wide temperature range to the glass transition state is necessary. In this study, the surrounding temperature was fixed at 40 °C to ensure sufficient light-absorption time.

## Additional Information

**How to cite this article**: Lee, Y. *et al.* Sequential Folding using Light-activated Polystyrene Sheet. *Sci. Rep.* **5**, 16544; doi: 10.1038/srep16544 (2015).

## References

[b1] Liu Y., Boyles J. K., Genzer J. & Dickey M. D. Self-folding of polymer sheets using local light absorption. Soft Matter, 8, 1703–2044 (2012).

[b2] Liu Y., Miskewicz M., Ecsuti M. J., Genzer J. & Dickey M. D. Three-dimensional folding of pre-strained polymer sheets via absorption of laser light. J. Appl. Phys., 115, 204911 (2014).

[b3] Liu Y., Mailen R., Zhu Y., Dickey M. D. & Genzer J. Simple geometric model to describe self-folding of polymer sheets. Phys. Rev. E, 89, 042601 (2014). 10.1103/PhysRevE.89.042601 24827268

[b4] Ryu J., D’Amato M., Cui X., Long K. N. & Qi H. J. Photo-origami-bending and folding polymers with light. Appl. Phys. Lett., 100, 161908 (2012).

[b5] Meng H. & Li G. A review of stimuli-responsive shape memory polymer composites, polymer, 54, 2199–2221 (2013).

[b6] Onal C. D., Wood R. J. & Rus D. Towards printable robotics: Origami-inspired planar fabrication of three-dimensional mechanisms. In Proc. IEEE Intl. Conf. on Robotics and Automation(ICRA), 4608–4613 (2011).

[b7] Felton S., Tolley M., Demaine E., Rus D. & Wood R. A method for building self-folding machines. Science, 345, 644–646 (2014). 25104380 10.1126/science.1252610

[b8] Shin B., Felton S. M., Tolley M. T. & Wood R. J. Self-assembling sensors for printable machines. In Proc. IEEE Intl. Conf. on Robotics and Automation (ICRA), 4417–4422 (2014).

[b9] Vaccaro P. O., Kubota K., Fleischmann T., Saravanan S. & Aida T. Valley-fold and mountain-fold in the micro-origami technique. Microelectronics Journal, 34, 447–449 (2003).

[b10] Bassik N., Stam G. M. & Gracias D. H. Microassembly based on hands free origami with bidirectional curvature. Appl. Phys. Lett., 95, 091901 (2009). 10.1063/1.3212896 PMC2752473 19787072

[b11] Small W. IV., Singhal P., Wilson T. S. & Maitland D. J. Biomedical applications of thermally activated shape memory polymers. J. Mat. Chem. 20, 3356–3366 (2010). 10.1039/B923717H PMC3023912 21258605

[b12] Guo X. *et al.* Two-and three-dimensional folding of thin filme single-crystalline silicon for photovoltaic power applications. P. Natl. Acad. Sci. USA., 106, 20149–20154 (2009). 10.1073/pnas.0907390106 PMC2781057 19934059

[b13] Cho J. H., Hu S. & Gracias D. H. Self-assembly of orthogonal three-axis sensors. Appl. Phys. Lett. 93, 043505 (2008).

[b14] Fernandes R. & Gracias D. H. Self-folding polymeric containers for encapsulation and delivery of drugs. Adv. Drug Deliver. Rev., 64, 1579–1589 (2012). 10.1016/j.addr.2012.02.012 PMC3462897 22425612

[b15] Stoychev G., Zakharchenko S., Turcaud S., Dunlop J. W. & Ionov L. Shape-programmed folding of stimuli-responsive polymer bilayers. ACS Nano, 6, 3925–3934 (2012). 22530752 10.1021/nn300079f

[b16] Felton S. M. *et al.* Self-folding with shape memory composites. Soft Matter 9, 7659–7694 (2013).

[b17] Gracias D. H. Stimuli responsive self-folding using thin polymer films. Chem. Eng. 2, 112–119 (2013).

[b18] Tolley M. T. *et al.* Self-folding shape memory laminates for automated fabrication, In Proc. IEEE/RSJ Intl. Conf. on Intelligent Robots and Systems (IROS), 4931–4936 (2013).

[b19] Tolley M. T. *et al.* Self-folding origami: shape memory composites activated by uniform heating. Smart. Mater. Struct., 23, 094006 (2014).

[b20] Hawkes E. *et al.* Programmable matter by folding. Proc. Natl. Acad. Sci. USA., 107, 12441–12445 (2014). 20616049 10.1073/pnas.0914069107 PMC2906559

[b21] Sliverberg J. L. *et al.* Origami structures with a critical transition to bistability arising from hidden degrees of freedom, Nature Mater. 14, 389–393 (2015). 25751075 10.1038/nmat4232

[b22] Zhang Q., Yan D., Zhang K. & Hu G. Pattern transformation of heat-shrinkable polymer by three-dimensional (3D) printing technique, Sci. Rep. 5, 8936 (2015). 25757881 10.1038/srep08936 PMC4355736

[b23] Lee D. Y., Kim S. R., Kim J. S., Park J. J. & Cho K. J. Fabrication of origami structure using pattern enclosed composite (PEC). In Proc. Intl. Conf. on Control, Automation and System (ICCAS), 313–315 (2013).

[b24] Miyashita S., Meeker L., Tolley M. T., Wood R. J. & Rus D. Self-folding miniature elastic electric devices. Smart Mater. Struct, 23, 094005 (2014).

[b25] Komolprasert V., Diel T. & Sadler G. Gamma irradiation of yellow and blue colorants in polystyrene packaging materials. Radiat. Phys. Chem., 75, 149–160 (2006).

[b26] Juhl T. B., Christiansen J. C. & Jensen E. A. Mechanical testing of polystyrene/polystyrene laser welds. Polym. Test. 32, 475–481 (2013).

[b27] Chivers R. A., Bonner M. J., Hine P. J. & Ward I. M. Shape memory and stress relaxation behavior of oriented mono-dispersed polystyrene. Polymer, 55, 1055–1060 (2014).

[b28] Sastri V. R. Plastics in medical devices: Properties, Requirements and Applications 2nd edn, Elsevier (2014).

[b29] Emblem A. Packaging technology, Fund. Mat. Process, 287–309 (2012).

[b30] Zhao X. M., Xia Y., Schueller O. J. A., Qin D. & Whitesides G. M. Fabrication of microstructures using shrinkable polystyrene films. Sensor. Actuator. A-phys., 65, 209–217 (1998).

